# Supplementary material for: Molecular Pharming of the Recombinant Protein hEGF-hEGF Concatenated with Oleosin Using Transgenic Arabidopsis
Source: Genes (Basel). 2020 Aug 19;11(9):959. doi: 10.3390/genes11090959 (PMC7564230; doi:10.3390/genes11090959)
Supplement: Supplementary file 1 [file genes-11-00959-s001.pdf]

Molecular pharming of the recombinant protein hEGF-hEGF concatenated with oleosin using transgenic *Arabidopsis*

Table 1. Expression analysis of fusion protein in transgenic *Arabidopsis* seeds.

	Line 1	Line 2	Line 3	Line 4	Line 5	Line 6
**Grayscale value**	37061.86741	10542.71198	98074.61904	10500.66634	10102.8499	8963.089624
**Quality（The mass of EGF in 8uL oil body suspension (ng)）**	43.2144	10.4168	118.672	10.3648	9.8728	8.4632
**Concentration(ng/uL)**	5.4018	1.3021	14.834	1.2956	1.2341	1.0579

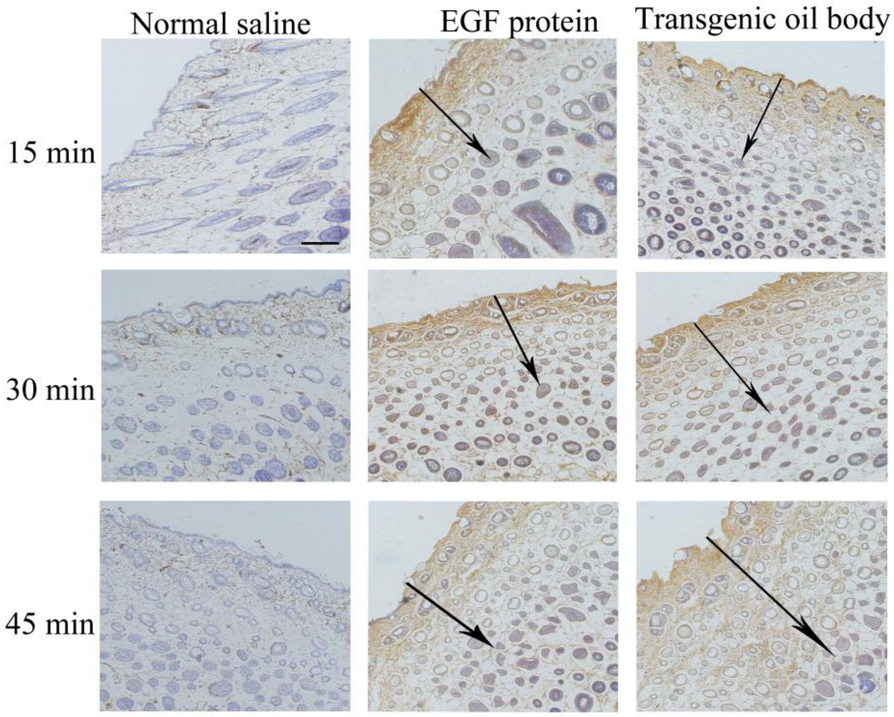

Figure 1. The ability of transgenic oil bodies bound hEGF to infiltrate into skin. The immunohistochemistry analysis of drug absorption at different times is shown. EGF protein is the positive group and Normal saline is the negative control group. The images were shown at 40×10 magnification (Scale bars = 50μm).
